# Resonating with Cellular Pathways: Transcriptome Insights into Nonthermal Bioeffects of Middle Infrared Light Stimulation and Vibrational Strong Coupling on Cell Proliferation and Migration

**DOI:** 10.34133/research.0353

**Published:** 2024-04-30

**Authors:** Xingkun Niu, Zhongyu Wu, Feng Gao, Shaojie Hou, Shihao Liu, Xinmin Zhao, Liping Wang, Jun Guo, Feng Zhang

**Affiliations:** ^1^ Quantum Biophotonic Lab, Key Laboratory of Optical Technology and Instrument for Medicine, Ministry of Education, School of Optical-Electrical and Computer Engineering, University of Shanghai for Science and Technology, Shanghai 200093, China.; ^2^ Wenzhou Institute, University of Chinese Academy of Sciences, Wenzhou 325001, China.; ^3^ Department of Nuclear Medicine, The First Affiliated Hospital of Shandong First Medical University and Shandong Provincial Qianfoshan Hospital, Jinan 250013, China.; ^4^ School of Radiology, Shandong First Medical University and Shandong Academy of Medical Sciences, Jinan 250024, China.; ^5^ The School of Biomedical Engineering, Guangzhou Medical University, Panyu District, Guangzhou 511436, China.

## Abstract

Middle infrared stimulation (MIRS) and vibrational strong coupling (VSC) have been separately applied to physically regulate biological systems but scarcely compared with each other, especially at identical vibrational frequencies, though they both involve resonant mechanism. Taking cell proliferation and migration as typical cell-level models, herein, we comparatively studied the nonthermal bioeffects of MIRS and VSC with selecting the identical frequency (53.5 THz) of the carbonyl vibration. We found that both MIRS and VSC can notably increase the proliferation rate and migration capacity of fibroblasts. Transcriptome sequencing results reflected the differential expression of genes related to the corresponding cellular pathways. This work not only sheds light on the synergistic nonthermal bioeffects from the molecular level to the cell level but also provides new evidence and insights for modifying bioreactions, further applying MIRS and VSC to the future medicine of frequencies.

## Introduction

The light–matter interaction can normally cause resonant absorption or resonant coupling phenomena when they share equal energy levels or frequencies [1], but the difference is whether the 2 systems, i.e., light and matter, exchange energies. For biosystems, resonant absorption means biomolecules absorb the energy from light without returning it back, i.e., single direction energy transfer, otherwise belongs to resonant coupling if energies can be exchanged between light and biomolecules in an oscillating manner, i.e., bidirectional energy exchange. Besides the radiation influence on evolution as critical drivers of biodiversity [2], the application of resonant absorption can be tracked back to the Nobel Prize in 1903, for Niels Ryberg Finsen’s contribution to the treatment of skin diseases with light radiation [3]. Later, resonant absorption has been widely extended to the biomedicine field, e.g., phototherapy [4,5], middle infrared stimulation (MIRS) [6–8], and even optogenetics [9]. Different from the earlier biomedical applications with wide-frequency-ranged resonant absorption, e.g., modifying cellular signal pathways [10,11] and promoting cell proliferation [12,13], recent advances pay more attention to MIRS with precise frequencies [14]. For instance, 44.0-THz light has been calculated to markedly lower down the unwinding temperature of double-stranded DNAs (dsDNAs) [15], and 53.5-THz MIRS was employed to improve the efficiency of polymerase chain reaction (PCR) [7,8] and drive the coassembly of DNA origami [16], as well as to regulate the activity of K^+^ ion channel thus influence neuronal signaling and zebra fish behavior [6]. Notably, MIRS does not like optogenetics requiring modifying animal genetic information but holds great potential to control animal behaviors, which has been demonstrated by more and more researches from cellular levels [17–19] to animal levels [20–22]. Nevertheless, there still remains an unknown gap between molecular levels and cell levels, since there are a large number of molecules containing identical chemical structures that can resonantly receive MIRS, but the synergetic and coupling reactions in cells can make it more complicated. In this sense, transcriptome sequencing should be a useful tool to fill up this gap, at least for the correlation between molecular genes and cellular pathways [17].

Along with the gradual penetration of quantum theory to biomedicine and the rapid development of terahertz sources [23,24], a new nomenclature “vibrational strong coupling” (VSC), as a typical tool of resonant coupling, has already been demonstrated its powerful role in the biochemistry field [25,26]. In the optical microcavity, the limited electromagnetic mode interacts reversibly with the collective macroscopic molecular vibrational polarization, and the strong coupling between the electromagnetic field and the molecule will produce a mixed state of mixed molecular and photon properties, namely, the molecular polarization excited state. Notably, VSC does not require any external energy input (e.g., laser), which is the most interesting point (i.e., can even occur in the dark) and dramatically differs from MIRS. A number of studies have demonstrated that the strong coupling mechanism can effectively modulate the intermolecular energy conversion process, thus affecting the kinetic behavior and product distribution of chemical reactions. Pioneered by Ebbesen et al., kinetics of bioreactions have been investigated within the optical resonators, e.g., bioreactions catalyzed by pepsin [27], adenosine triphosphatase [28], DNA polymerase [29], sucrase [30], and coassembly of biomolecules, e.g., DNA origami [31], and the results show that these reaction efficacies can be substantially modified by the VSC effects. Because of its potential and powerful applications in the medicine, especially medicine of frequencies, VSC has attracted intensive attention from interdisciplinary researchers. However, VSC has scarcely been studied on cellular levels yet, though its cavity volume fits for cell culture.

**Fig. 1.**
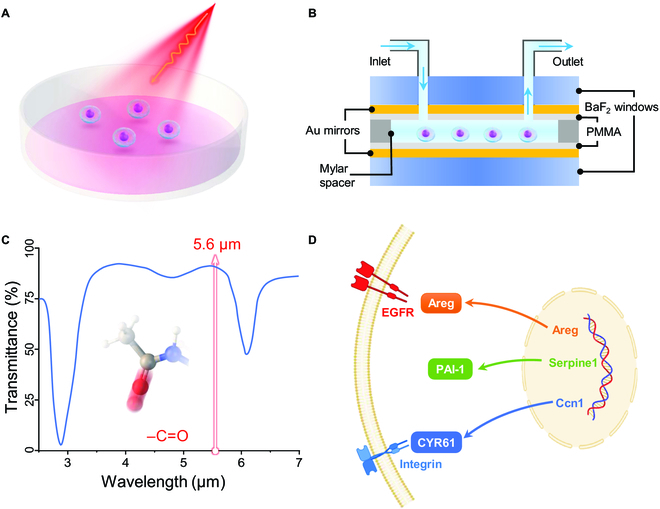
Schematic mechanisms for the nonthermal bioeffects of MIRS and VSC on cells. (A) Schematic MIRS setup for the fibroblast cell resonant absorption. For real setup, please refer to Fig. S1. (B) Configuration of the FP cavity for studying VSC effects on fibroblasts. For real setup, please refer to Fig. S2. (C) MIRS induces the resonant absorption of carbonyl group that avoids the water absorption at 5.6 μm. (D) Schematic cellular pathways related to the cell proliferation and migration, including the main genes: Areg, Serpine1, and Ccn1.

Herein with the strong curiosity, we systematically compared nonthermal bioeffects of both MIRS (Fig. 1A) and VSC (Fig. 1B) at the same resonant frequency 53.5 THz (5.6 μm, by a quantum cascade laser; Fig. 1C) on cellular pathways regarding to both cell proliferation and cell migration (Fig. 1D). The results showed that both the proliferation rate and the migration ability of fibroblasts are substantially promoted under a short-term MIRS or VSC treatments. The specific nonthermal bioeffects of MIRS and VSC on fibroblasts at the molecular level were comprehensively analyzed by transcriptome sequencing. This work could pave the way toward the medicine of frequencies, e.g., precisely regulating cell functions or behaviors through photon-energy-injection-based resonant absorption or just place the biosystem in an optical resonator with a special cavity distance.

## Results and Discussion

**Fig. 2.**
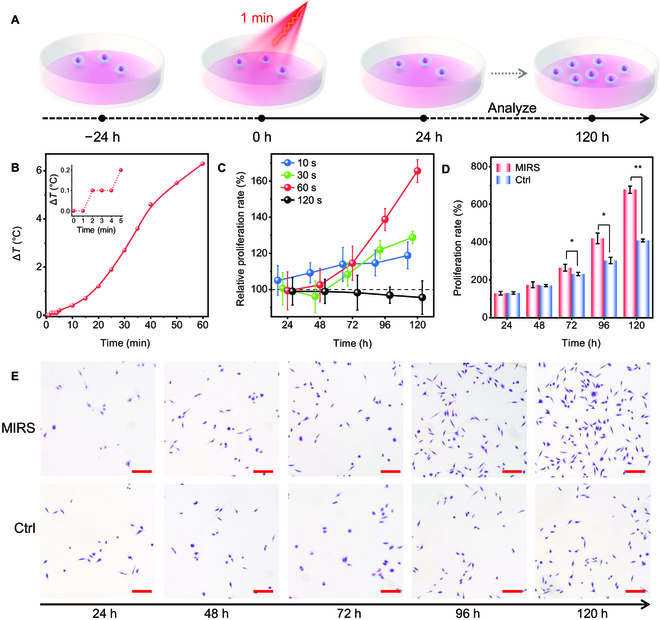
MIRS enhances cell proliferation. (A) Schematic of cells experiencing MIRS and analysis. (B) Dependency of temperature variation of cell culture media on the MIRS time. Inset is a zoom-in figure. (C) Plots of the relative cell proliferation rate against the culture time after MIRS with different time. (D) Comparison of cell proliferation rate at different culture time after 1-min MIRS, Ctrl represents there was no MIRS treatments. (E) Cell proliferation images taken at different times after 1-min MIRS. Cell culture was fixed at the time of detection and stained with crystal violet. Scale bars are all 200 μm. The fibroblasts used were all L929 cells.

The energy level of 53.5-THz light corresponds to the stretching vibration of carbonyl groups [6], which are abundant in cytokines, regulatory proteins, and DNA molecules. The resonant absorption of MIRS by carbonyl groups equals to inject additional pure photon energy into biomolecules, which inevitably enhance the vibrational amplitude and thus their participated bioreactions. With the high transmission of 5.6-μm MIR to the water in cells and culture media, the working power density in our following MIRS experiments (Fig. 2A) can reach up to 1 W/cm^2^, which is almost 1,000 times powerful than that used in earlier wide-range frequency-based MIRS experiments [12]. To check out the thermal effect, the temperature of cell media with different MIRS time was monitored. The results showed that the temperature increase was not detectable if the MIRS time was less than 1 min, and no more than 0.1 °C even if extending MIRS to 4 min (Fig. 2B).

Upon absorption of the photon energy by biological tissue, gasification and mechanical waves are produced if the energy density surpasses a certain threshold. If the energy density is below this threshold, only mechanical waves will be generated. Therefore, the optimal approach for laser control would involve irradiating at an energy density that falls beneath the gasification threshold. The cell viability under different exposure times shows there was no noticeable change in cell activity compared to the control (Ctrl) group during the 1-min MIRS (Fig. S3). With this knowledge, we next investigated the dose dependency of MIRS on relative cell proliferation rate. To this end, we define 2 functions as: (a) the proliferation rate, indicating the capacity to proliferate, can be determined by the ratio of the cell viability on day *N* after MIRS/VSC treatments to the cell viability immediately after MIRS; (b) the relative proliferation rate, representing the growth rate changes, is calculated by the ratio of the cell viability on day *N* after MIRS treatments to the cell viability on the first day (*N* = 1) before MIRS.

In comparison, the relative proliferation rates at 120 h were 156%, 128.7%, and 118.8% with MIRS time of 1 min, 30 s, and 10 s, respectively (Fig. 2C). However, we found that the relative proliferation rate in the 2-min MIRS group was less than 1, that is, cell proliferation was inhibited after 2-min MIRS (Fig. 2C). However, 2-min MIRS only increased the cell environment by 0.1 °C; thus, we can determine that entirely nonthermal influences, excluding thermal effects, could also have detrimental impacts on cells. Therefore, within the duration of nonthermal effects (<4 min), we further adjust the MIRS time to stay within the range that promotes cell health (<2 min). Based on the above results, we chose 1-min MIRS for all following experiments in order to completely exclude the thermal effects. Clearly, cells showed stronger proliferative ability at 72 h after 1-min MIRS compared with the Ctrl group (Fig. 2D). The proliferation rate increased by 268.5% at 120 h, which can be clearly judged from the density statistics (Fig. S4) and the microscopic photos as well (Fig. 2E), indicating MIRS substantially enhancing cell proliferation.

**Fig. 3.**
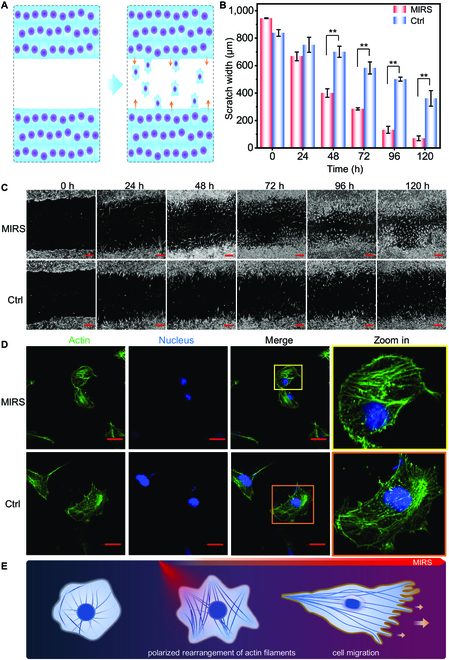
MIRS enhances cell migration. (A) Schematic principle of the scratch assay for measuring cell migration ability. A blank area was created by scratching the cell monolayer, which was gradually recovered (healed) mainly by cell migration from the edge of the scratch. Cell migration ability is assessed by the scratch width variation to time. (B) The average width of cell scratch area dependency on the post-MIRS time. (C) Microscopy images for the dependency of cell scratch area on the post-MIRS time after 1-min 5.6-μm MIRS. Scale bars represent 60 μm. (D) Fluorescence images of cellular actins for morphological comparison between fibroblasts at post-MIRS time of 120 h with and without 1-min 5.6-μm MIRS. Scale bars represent 20 μm. (E) Cartoons for enhancing cell migration ability through remodeling the polarity of actin filaments.

Next, the cell migration was examined on the same MIRS setup with utilizing an in vitro wound healing assay to analyze the migratory response of cells. The cell migration ability was assessed by comparing the recovery degree of scratched areas of cell monolayer on dishes, which was measured at 120 h after MIRS (Fig. 3A). Results showed that the difference of cell migration abilities between MIRS groups and the Ctrl group can be distinguished after 48 h (Fig. 3B). Regarding the width of the scratch area, the proportions of cells covering the scratched areas varied notably between the MIRS group and the Ctrl group. In comparison of the width of the scratch area at 120 h, the proportions for cells covering the scratch areas were 92.5% and 56.9% for the MIRS group and Ctrl, respectively (Fig. 3B and C), indicating that MIRS markedly enhanced the migration ability of fibroblasts. These results allude to the beneficial impact of MIRS in enhancing the migration ability of fibroblasts thereby accelerating the wound healing process.

Cell migration is a mechanical movement supported by the cytoskeleton, whose changes can be reflected by the tempospatial arrangement of actin [32]. The cell morphology at 24 h after MIRS was quantitatively analyzed by fluorescence microscopy, which showed that the actin filaments are arranged in a unidirectional polar state (Fig. 3D), indicating a polarity remodeling initiation of the fibroblast skeleton after MIRS. The contraction of actin filaments efficiently directs energy into unidirectional cell displacement, with stress fibers enhancing this movement by becoming more organized and homogenous, leading to increased cellular migration efficiency due to the cell’s polarized structure (Fig. 3E). Cell migration requires the development of spatial asymmetry, enabling the conversion of internal forces into a net shift of the cell body, as evidenced by the distinct front and rear of the cell. Fluorescence staining showed that the overall cell size after MIRS was much longer, evidenced by the larger axial-diameter ratio and more asymmetric cell morphology (Fig. S5).

**Fig. 4.**
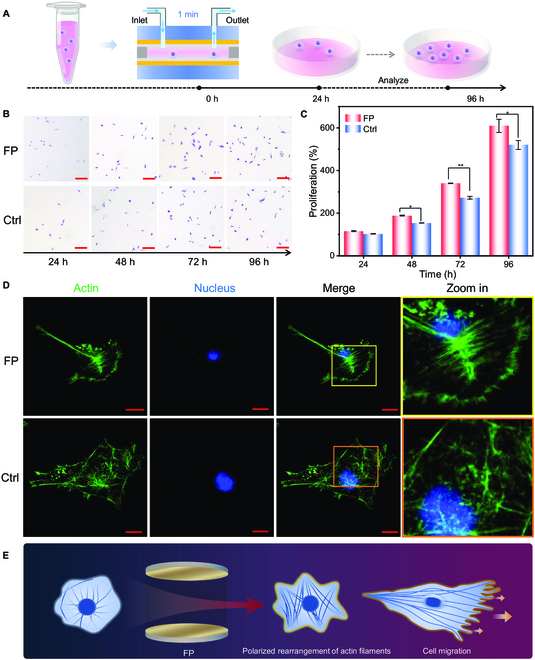
VSC enhances cell proliferation and migration. (A) Schematic of cells experiencing VSC (for real setup, please refer to Fig. S2) and analysis. (B) Cell images taken at different proliferation times after incubation in FP cavities. Cell culture was fixed at the time of detection and stained with crystal violet. Scale bars represent 200 μm. (C) Comparison of time-dependent cell proliferation under different culturing conditions. (D) Fluorescence images of cellular actins for morphological comparison of fibroblasts between in and outside of FP cavities. Scale bar represents 10 μm. (E) Cartoons for actin filament alteration after VSC treatment and finally influencing cell migration.

In contrast to MIRS, VSC relies on the resonant coupling between molecular vibrations and optical resonator modes. Since there is no external energy input to the resonator, i.e., here occurs ground-state chemical reaction under VSC, in contrast to the excited-state reactions in which the reactants are in a higher-energy state achieved through external energy input of heat or light, VSC can drive inside reactions even in the dark [31,33]. The average size of fibroblast cell nucleus is 10 μm, so the cell suspension can easily flow through Fabry–Pérot (FP) cavity without mechanical damage (Fig. S2). The resonant coupling between cells and the resonator can be realized by measuring the free spectra range (FSR) of optical frequency combs (Fig. S6). For VSC treatment of cells within the microcavity (Fig. 4A), we observed a distinct increase in the proliferation ability of the suspended cells (Fig. 4B and C). This was further evidenced by fluorescent staining, highlighting a restructuring of actin filaments akin to the trends observed in MIRS case (Fig. 4D). Based on fluorescence staining results, it showed that the arrangement of actin filaments after VSC incubation leans toward forming more single bundles (Fig. 4E). Cells after VSC treatments showed similar morphological changes as those treated with MIRS. To assess whether VSC treatment enhances fibroblasts migration capabilities, we conducted Transwell migration assays to investigate the alterations in cell migration following passage through the FP cavity. According to the results (Fig. S11B), 48 h after VSC treatment, more cells migrated from the upper chamber to the lower chamber of Transwell, indicating that an improvement in their migration capacity. This observation substantiates our hypothesis that VSC can indeed facilitate cell migration, as inferred from morphological changes observed in the cells. In this case, the Ctrl group was the cells treated after the cavity with the electromagnetic field removed (the metal reflector removed). In order to verify whether the experimental results were affected by the container effect of the cavity, we also monitored the proliferation and migration ability of the cells waiting and treated in the extracellular centrifugation tube under the same environment during the experiment (Ctrl*). According to the results (Fig. S11), we can assume that the enhancement of cell proliferation and migration after FP cavity treatment is the effect of VSC.

**Fig. 5.**
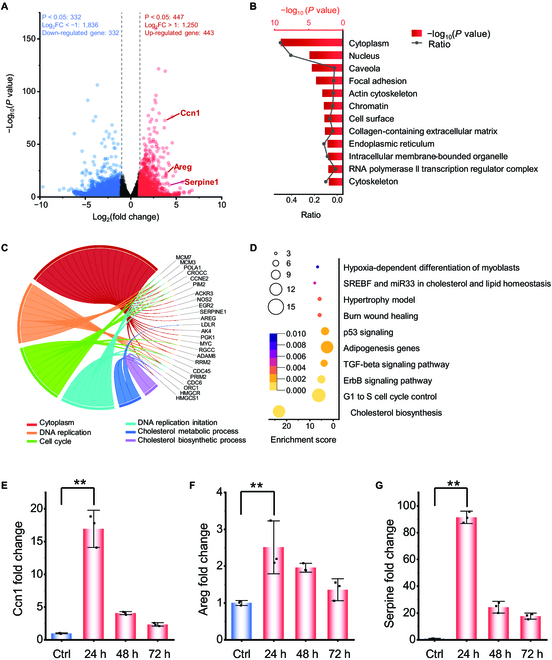
Transcriptomic sequencing of MIRS-treated cells. (A) Identification of significantly differentially expressed genes after MIRS. (B) GO enrichment statistics. The histogram and plot line show the significance and the proportion of differentially expressed genes corresponding to each cell component term, respectively. (C) Chorography displays the interconnectivity between differentially expressed genes and GO enrichment terms. (D) Signaling pathways that could be influenced by MIRS. The color and size of bubbles represent the significance and the number of differentially expressed genes associated with the corresponding pathways, respectively. Alterations of (E) Ccn1, (F) Areg, and (G) Serpine1 genes after 1-min 5.6-μm MIRS and extending incubation for another 72 h. All data obtained from fibroblasts within 72 h after 1-min 5.6-μm MIRS.

To figure out the molecular mechanism of MIRS enhancement of cell proliferation and migration (Fig. S7), we further employed transcriptome sequencing to check the whole-genome variation of cells at 24 h after MIRS treatment. With differentially transcribed genes after MIRS, we analyzed which biological processes regulate cell proliferation and migration and which cellular components or molecular functions are altered. The differential gene results showed that 443 genes were significantly up-regulated and 332 genes were significantly down-regulated in fibroblasts after MIRS (Fig. 5A). The altered transcription of genes after MIRS suggests changes in the proteins coded by these genes and their respective functions. We categorized all these transcription-altered genes according to Gene Ontology (GO, an extensive global repository for deciphering the biological implications of genes), conducting a thorough comparison of enrichment and significance. This helped identify the most probable molecular functions, cellular components, or biological processes impacted at 24 h after MIRS. From the aspect of bioinformatics, GO enrichment analysis of differentially expressed genes [34,35] reveals the expression of genes related to biological processes such as DNA replication and cell cycle, which are closely related to the cell proliferation, while the differential genes for cell migration are related to actin cytoskeleton, adhesion plaques, and collagen. These genes consist with our experimental results, particularly the observed increase in cell migration ability.

Then, we identified the genes that show up-regulated transcription related to these specific GO terms of interest (Fig. 5B). Given the extensive number of up-regulated genes, the string diagram is designed to help us identify the most crucial genes influenced by MIRS. Each of these up-regulated genes corresponds to one or more GO terms. We performed detailed statistics to establish a one-to-one correlation between significantly up-regulated genes and the GO terms of interest. Genes involved in cell cycles, DNA replication, and cholesterol synthesis are differentially transcribed (Fig. 5C). A large number of differential genes involved in transforming growth factor-β (TGF-β), erythroblastic oncogene B (ErbB), and phosphatidylinositol 3-kinase-protein kinase B (PI3k-Akt) signaling pathways were highly transcribed after MIRS, which are responsible for promoting cell proliferation and migration by regulating the related enzymes and growth factors [36–38]. Signaling pathways related to wound healing, muscle hypertrophy, and cholesterol synthesis were activated as well, all suggesting the nonthermal bioeffect of MIRS on the fibroblast proliferation and migration (Fig. 5D).

According to the results of signal pathway enrichment analysis, TGF-β, ErbB, and PI3k-Akt, which are the 3 signaling pathways involved in cell proliferation and migration, are most likely the signaling pathways affected by MIRS. Through the differentially transcribed genes corresponding to these 3 signaling pathways, we selected 3 highly correlated up-regulated genes: the protein encoded by amphiregulin (Areg) is an important ligand of the receptor tyrosine kinase epidermal growth factor receptor (EGFR) (ErbB1/HER1) in the ErbB signaling pathway. When Areg binds to EGFR, it triggers the activation of EGFR kinase activity. After EGFR activation, it regulates cell proliferation, migration, and survival by activating multiple downstream signaling pathways, such as mitogen-activated protein kinase, PI3K/Akt, etc. [39–41]. Ccn1 encodes the extracellular matrix protein cysteine-rich angiogenic inducer 61 (Cyr61), which regulates cell proliferation, migration, and other functions. Previous researches showed that PI3K/Akt pathway was involved in the regulation of Ccn1 induced cell migration [42–44]. Serpin family E member 1 (Serpine1), which is an important gene in fibroblasts cell migration and involved in multiple stages of skin repair, and its encoded protein plasminogen activator inhibitor-1 can regulate cell migration by altering the proteolytic microenvironment around cells. TGF-β signaling pathway can regulate the expression of SERPINE1 gene and increase the synthesis of plasminogen activator inhibitor-1 [45,46].

To further confirm the transcriptional results, we performed real-time fluorescent quantitative PCR of these 3 genes on the cellular level at different periods after MIRS. From the results, we can see that the transcription of these 3 genes was noticeably increased at 24 h (Fig. 5E to G). In combination with the experimental results of cell proliferation and migration, we can conclude that MIRS markedly improved the expression of 3 genes. The corresponding expressed proteins encoded by these genes can activate the signaling pathways related to cell proliferation or migration, eventually enhancing the proliferation and migration of cells through regulating the fibroblast growth factors. At 48 and 72 h, the amount of transcription of these 3 genes gradually decreased, indicating that the bioeffect of MIRS on cells was not permanent and can gradually recover. In addition, through GO enrichment analysis, we know that DNA unwinding, actin proteins, and adhesion plaques may play important roles in the regulation of proliferation and migration of fibroblasts. Therefore, the enhancement effects of MIRS on proliferation and migration is a subsequent effect that occurs after activation of the relevant pathways, which explains that the regulation of cell proliferation becomes significant only at 72 h after MIRS. In addition, the regulation of cell migration after MIRS has an obvious effect from the start. This is due to the different time required for pathway activation to produce effects on proliferation and migration. The MIRS is used to target the common carbonyl group in the cell; therefore, other cellular pathways or components could be also influenced by MIRS, which makes more complex regulation in the cell. This could explain the above 2-min MIRS of cells without damage but inhibited growth.

Compared with MIRS, transcriptome sequencing was also performed on cells at 24 h after VSC treatments (Fig. S8). We performed transcriptome sequencing on cells 24 h after VSC regulation in the FP cavity to compare the transcriptome sequencing results of the VSC-treated group at the same time after MIRS regulation. The approach to identifying the influence pathway involves focusing on key genes based on their associated biological functions as recorded in the database. We also analyzed the differential transcription genes of cells after VSC treatment using both the GO and Wikipathways databases to determine if the regulation mechanism of VSC on cells aligns with that of MIR irradiation. The analysis revealed that a more complex set of differentially transcribed genes appeared in VSC-treated cells, so the GO enrichment analysis of gene-related functions also revealed more class items (Fig. S8A). Through transcriptome sequencing of the cells treated with both methods, we observed notable findings in significantly enriched categories. In the enrichment analysis using both databases, TGF-β signaling pathway and ErbB signaling pathway showed high enrichment, which was very consistent with our previous analysis of the cellular regulatory mechanism of MIRS irradiation. Therefore, we compared the transcriptional differences of the first 3 genes after the 2 regulation modes, and the results showed that Areg, Ccn1, and Serpine1 all up-regulated the transcription, particularly at 24 h after VSC treatments (Fig. S9), indicating that these 3 genes and related pathways play an important role in enhancing cell proliferation and promoting actin recombination by VSC. It also confirms our previous analysis of the cellular mechanism of MIRS regulation.

Both intramolecular chemical bonds and biological macromolecules, such as proteins and nucleic acids, possess distinct vibrational frequencies. Under VSC conditions, external vibrations interact with these molecular frequencies, impacting their structures and functions. In the context of cell response to VSC within a cavity, 2 potential effects become evident. First, VSC intricately regulates the transcription and translation processes of genes, thereby affecting protein synthesis and overall cellular function. Secondly, within cells, both cellular structures and organelles, including the cytoskeleton and nuclear membrane, exhibit unique vibrational characteristics. External vibrations can induce mechanical changes within cells, consequently influencing signal transduction and gene expression. These progressively cascading effects extend to the intricate regulation of genomics and cellular function. The regulatory effects of MIRS and VSC on cells showed high overlap in cell function, morphology, and transcriptional genes. However, because there is no external energy injection, VSC cannot achieve the same high promotion effect as MIRS because of direct resonance absorption, but there is no need to worry about cell damage caused by energy exceeding the threshold. Both methods aim to study the regulation of cells by using the resonance effect of carbonyl vibration. What is more similar is that the regulation time of the 2 methods on cells is short in a few minutes, during which the resonance absorption of the carbonyl group to MIRS and the resonance coupling of the VSC to the carbonyl group increase the vibration of the carbonyl group in cells. After the initial reaction of a few minutes, the relaxation process of the carbonyl group returning to the original state produced secondary reactions such as changes in intracellular signaling pathways, which realized the regulatory effects such as the improvement of cell proliferation and migration ability in our experiments. This gives us confidence to further study the frequency regulation of organisms. By further refining the frequency and power selection of resonance absorption and further enhancing the effect of resonance, we can achieve better regulation effect of organisms.

## Conclusion

In summary, we have systematically investigated the resonant absorption and coupling with carbonyl groups in cells by MIRS and VSC. Results show that both proliferative and migratory abilities of fibroblasts were substantially improved by both methods. Through transcriptome sequencing, we further confirmed the differential transcription genes that fill up the gap for understanding the nonthermal resonant effects on the cellular level. In contrast, VSC can completely exclude the thermal effects on cells, being a strong control for MIRS at the same frequency. On the other hand, the bioeffects on cells of VSC are not that weaker in comparison to MIRS, though VSC completely relies on the inner energy of cells. These results will inspire us to deepen our understanding of the highly efficient utilization of energies in cells; meanwhile it could shed light on developing new techniques for biomedical fields, e.g., wound healing and repair, cancer therapies, and so on.

## Materials and Methods

### Cell culture

L929 mouse fibroblasts were purchased from American Type Culture Collection (Manassas, VA, USA). The cells were cultured in Dulbecco’s Modified Eagle Medium (Gibco, NYC, USA) supplemented with 10% fetal bovine serum (FBS) (Pricella, Wuhan, China) and 1% penicillin/streptomycin (Beyotime, Jiangsu, China) and maintained at 37 °C in a 5% CO_2_ environment with medium changed every 2 to 3 days.

### MIR laser emitter

A pulsed quantum cascade laser (QCL, 176-mW average, MonoLux-56) was used to emit a 5.6-μm MIR laser. The laser can be set in the emission wavelength range of 5.35 to 5.94 μm, a pulse-triggering frequency of 1 to 100 kHz, an output pulse width of 50 to 200 ns, and a rise/fall time of 5 ns. The output beam was nearly collimated with the lens inside the QCL package, with a divergence of 3 mrad in the vertical direction and 2 mrad in the horizontal direction. The laser was set to operate continuously at a wavelength of 5.6 μm, and the laser power was set with the distance of 100 mm from the laser aperture. The laser power was quantified with an optical power meter (Endurance Lasers, FL, USA), and the size of the laser spot was determined through the knife-edge method. Also, the calculated laser power density was 1 W/cm^2^.

### MIRS assay

L929 cell suspensions in 96-well plates (Titan, Shanghai, China) were allowed to adhere overnight before laser stimulation experiments. As proteins in the cell culture medium have strong absorption at 5.6 μm, the culture medium in the wells was replaced with phosphate-buffered saline (PBS) for the experiments with laser stimulation (including the Ctrl groups). The 96-well plate was placed under the laser aperture, and the wells were divided into experimental and control regions (Fig. S4). The cells in the experimental region were successively irradiated with a 5.6-μm laser, with the unirradiated wells in the plate covered with a light shield during stimulation. One well was irradiated at a time under ambient temperature. The cells in the control region of the 96-well plate received the same treatment as the experimental region, except for the laser stimulation.

### Cell viability assay

Cell viability was measured using the cell counting kit-8 assay. Cells were seeded in a 96-well plate at a density of 5 × 10^3^ cells/ml, with 200 μl of cell suspension per well. After laser stimulation, cell viability was measured in both experimental and Ctrl groups every 24 h. Cell counting kit-8 solution (10 μl; Yeasen, Shanghai, China) was added to each well, and the plate was incubated for 3 h before measuring the absorbance at 450 nm using a microplate reader (Synergy H450, BioTek).

### Cell migration assay

MIRS treatment: Cells were seeded in a 96-well plate at a density of 5 × 10^5^ cells/ml and cultured overnight to form a monolayer. A scratch wound was created on the monolayer and followed by the laser stimulation. The cell medium was replaced with the serum-free culture medium, and the scratch wound area was observed and photographed every 24 h under a microscope. The images were analyzed and processed using ImageJ software. VSC treatment: The cell suspension treated with FP cavity was collected and centrifuged separately from other control cells, then resuspended with the basic medium without FBS. The concentration of the 3 groups was adjusted to the same 5 × 10^5^ cells/ml. To each well of the 24-well plate, 500 μl of complete medium containing 10% FBS was added, followed by the addition of 200-μl cell suspension into the Transwell chamber. The setup was cultured for 48 h, after which the cells were fixed and stained with crystal purple. The results of cell migration in the 3 groups were observed by microscopy (Axio Vert. A1, Carl Zeiss).

### Fluorescence staining

After laser stimulation for 24 h, L929 cells in the experimental and Ctrl groups were subjected to immunofluorescence staining. The cytoskeleton of cells was labeled with iFluor 488 phalloidin (Yeasen Biotechnology), and the nucleus were labeled with 4ʹ,6-diamidino-2-phenylindole (Adamas Life, Shanghai, China). The cells were firstly fixed in 4% paraformaldehyde in PBS at room temperature for 10 min, then were permeabilized with 10% Triton X-PBS for 10 min, and then were stained for the cytoskeleton and nuclei. The images were observed and photographed using the inverted fluorescence microscope (Axio Vert. A1, Carl Zeiss) and the high-resolution confocal laser microscope (Nikon A1 5000) and processed using ZEN, NIS-Elements Viewer, and ImageJ software.

### FP cavity

The assembled parts of FP cavity are shown in Fig. S5. Two flat polished calcium fluoride windows (Fig. S5B) are fixed by plasma-sputtered gold layer and poly(methyl methacrylate) spin coating to make a reflector (Fig. S5C) with 70% reflectivity. A certain thickness of polyethylene terephthalate ring film is sandwiched between the 2 reflectors to form a reflection cavity. The reflection cavity formed by the stainless-steel cover plate and the base is clamped with the window and fixed with nuts to form a stable FP cavity structure. The spectrum of FP cavity under the infrared spectrometer is a series of resonance peaks (Fig. S6), and the relationship between cavity distance (*L*) and the average distance between adjacent resonance peaks (free spectra range, *FSR*) is as follows:

$$L=\frac{m\cdot 10^{4}}{2\eta \cdot FSR}$$

where *m* and *η* are the cavity mode and refractive index, respectively. According to the frequency of carbonyl stretching vibration (1,784.33 cm^−1^) and considering the size of cells, we modulated the cavity distance to 27.39 μm (*m* = 13) to ensure that cells can flow smoothly through the FP cavity and be collected without mechanical damage (Fig. S10).

### Cell treatment in FP cavity

The FP cavity for cell treatment is shown in Fig. S5D. After centrifugation, the cells were resuspended with PBS solution to achieve a concentration of 10^6^ cells/ml. The cell suspension was injected into the FP cavity through a peristaltic pump with a hose. One end of the FP cavity was the injection port, and the other was the output outlet. The peristaltic pump speed was set to make the cells flow slowly in the FP cavity, and the effective treatment time of the cells within the FP cavity was ~1 min on average. Cells cultured within the FP cavity flow out of the outlet and are collected into the centrifuge tube. The concentration of both the collected cells and the control cells was adjusted to equal the concentration at inoculation. The distinct control groups were established for comparison. During VSC treatment, the cells in the Ctrl group were also passed through the cavity without forming a reflective layer (the windows of the gold-plated layer were replaced by a fully penetrating window), and the cells in the Ctrl* group are slightly oscillated in the centrifuge tube to prevent adhesion. The whole device was placed in an environment of 37 °C and 5% CO_2_ during regulation to ensure that the cell state was not affected. All 3 groups of cells were treated in the same environment for an equivalent duration.

### Transcriptome sequencing

Cells were seeded in a 96-well plate at a density of 1 × 10^4^ cells/ml. After MIRS and VSC experiments, they were cultured for 24 h. The cells were collected and digested with 0.25% trypsin-EDTA (Gibco, NYC, USA). The RNA libraries were sequenced on the Illumina NovaSeq 6000 platform by OE Biotech, Inc., Shanghai, China. The differential gene expression was analyzed using the criteria of *P* value < 0.05 and |log_2_FC| > 1. Statistics analysis was performed by GO and Wikipathways.

### Real-time PCR

Total RNA was extracted using irzol reagent (GlpBio, CA, USA) and reverse transcribed into cDNA using the SPARKScript II RT Plus Kit (SparkJade, Shandong, China). Real-time PCR was performed with TB Green Premix Ex Taq (TaKaRa) on a LightCycler 480 II real-time PCR system (Roche, Basel, Switzerland) with 40 amplification cycles, and the relative transcript levels were compared by the ΔΔCt method. The primer list is provided in the supplementary table (Table S1). Glyceraldehyde phosphate dehydrogenase was used as the internal control. The reactions were conducted in triplicate.

## Data Availability

The data supporting the findings of this study are available within the article.
