# Supplementary material for: Resonating with Cellular Pathways: Transcriptome Insights into Nonthermal Bioeffects of Middle Infrared Light Stimulation and Vibrational Strong Coupling on Cell Proliferation and Migration
Source: Research (Wash D C). 2024 Apr 30;7:0353. doi: 10.34133/research.0353 (PMC11062510; doi:10.34133/research.0353)
Supplement: Supplementary 1 — Figs. S1 to S11 Table S1 [file research.0353.f1.docx]

**Supporting Information**

**Resonating with Cellular Pathways: Transcriptome Insights into Nonthermal Bioeffects of Middle Infrared Light Stimulation and Vibrational Strong Coupling on Cell Proliferation and Migration**

*Xingkun Niu^1,2,#^, Zhongyu Wu^3,4,#^, Feng Gao^2,#^, Shaojie Hou^1,2,5,#^, Shihao Liu^2^, Xinmin Zhao^1^, Liping Wang^2,^*, Jun Guo^2,^*, Feng Zhang^1,2,^**

^1^ Quantum Biophotonic Lab, Key Laboratory of Optical Technology and Instrument for Medicine, Ministry of Education, School of Optical-Electrical and Computer Engineering, University of Shanghai for Science and Technology, Shanghai 200093, China;

^2^ Wenzhou Institute, University of Chinese Academy of Sciences, Wenzhou 325001, China;

^3^ Department of Nuclear Medicine, The First Affiliated Hospital of Shandong First Medical University & Shandong Provincial Qianfoshan Hospital, Jinan 250013, China;

^4^ School of Radiology, Shandong First Medical University & Shandong Academy of Medical Sciences, Jinan 250024, China;
^5^ The School of Biomedical Engineering, Guangzhou Medical University, Panyu District, Guangzhou 511436, China.

**Supplementary Figures**


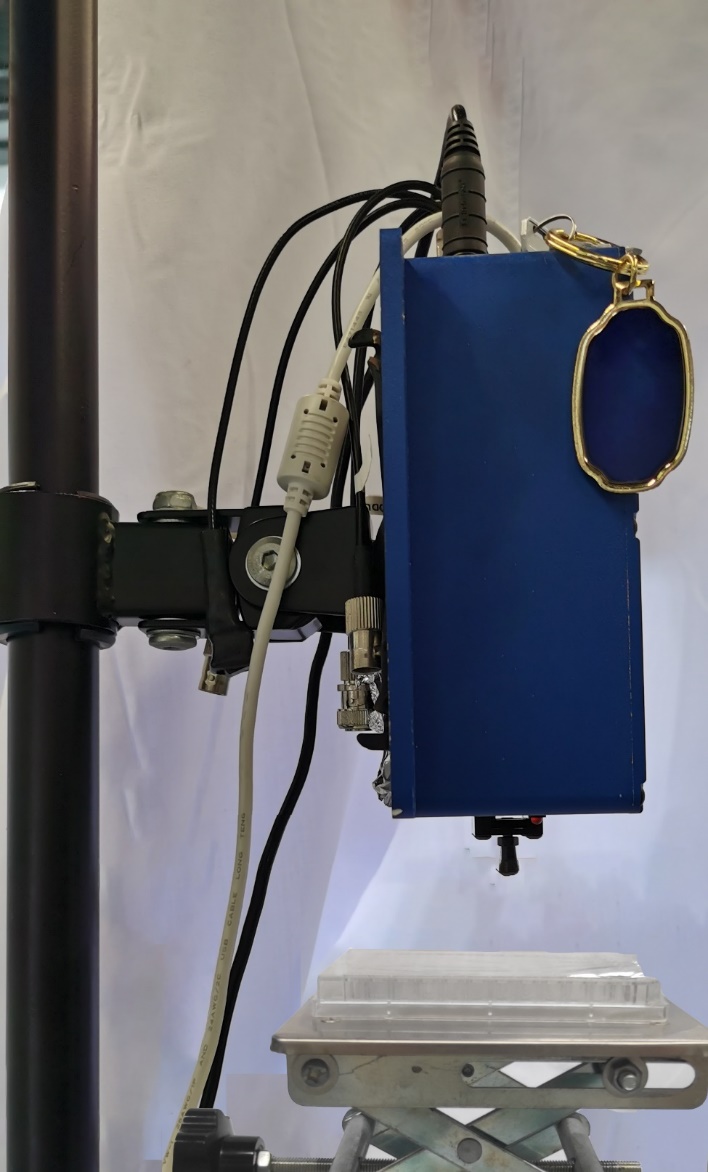


**Figure S1.** **Photo of MIRS device.** For MIRS, the cell culture plate was covered with a 1.5-μm thick mylar film, which ensured that the cells were in a sterile environment.

**Figure S2. Photos of FP cavity and the cell treatment setup.** a) The structure of the FP cavity device comprises, from left to right, the base, the lower gasket, the lower window, the spacer, the upper window, the upper gasket, the cover plate and the fixing nut. The calcium fluoride window b) was sputtered with gold films and then spun with PMMA to form a stable reflective and protective layer c). d) The cell suspension was injected into the FP cavity by peristaltic pump, and then exported to the centrifugal tube by peristaltic pump for collection.


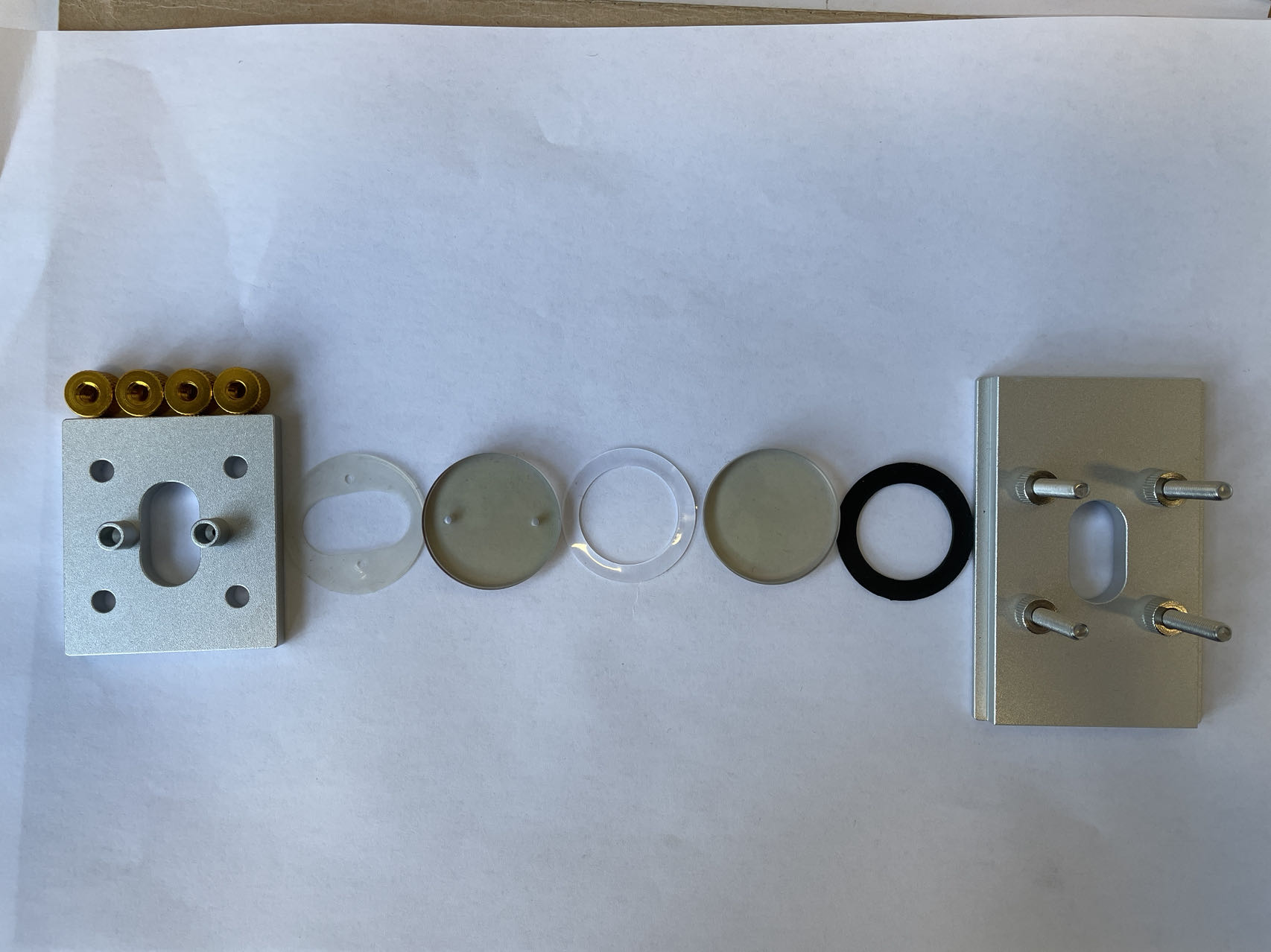


**a**


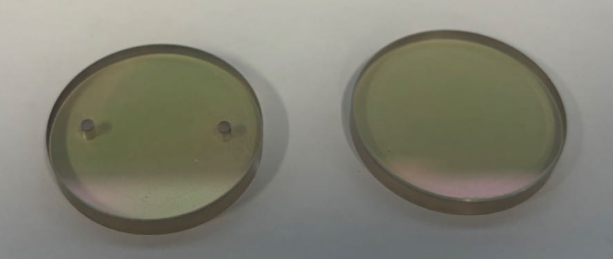

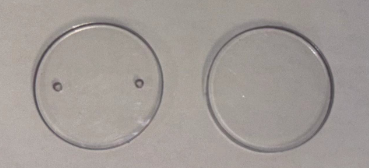

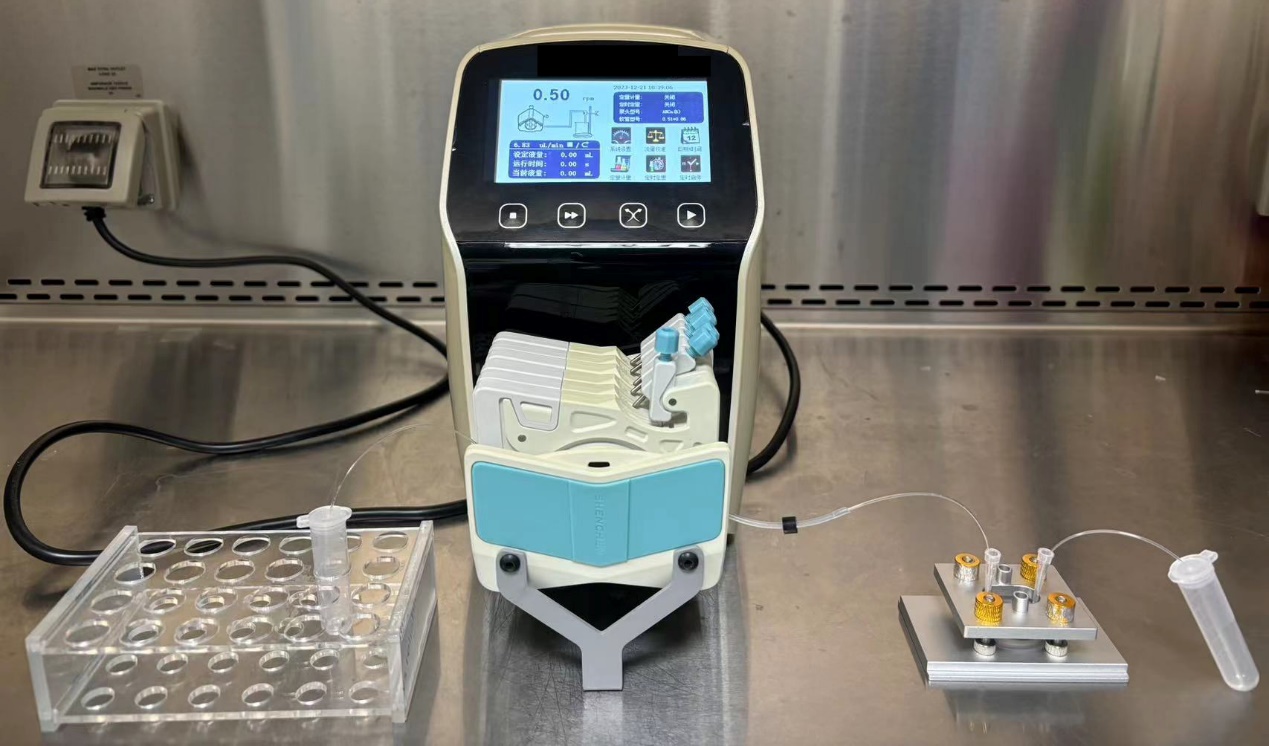


**b**

**c**

**d**

**
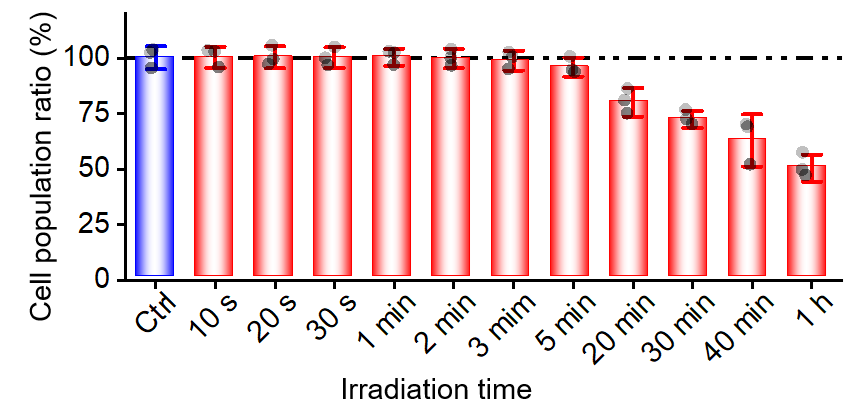
**

**Figure S3.** **Cell viability of fibroblasts after MIRS for different time.**


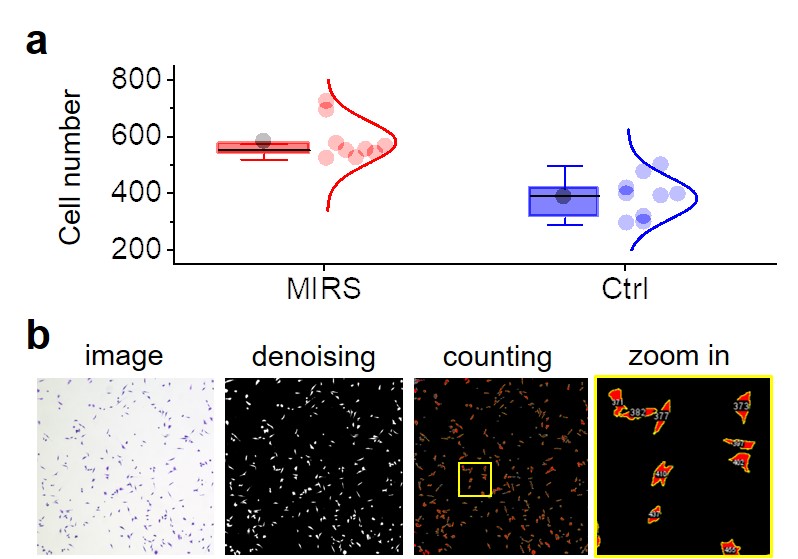


**Figure S4. MIRS enhanced fibroblast proliferation.** a) Statistical comparison of the number of cells at 120 h after 1-minute MIRS. b) Method for calculating the number of cells from the fluorescence microscopy images.


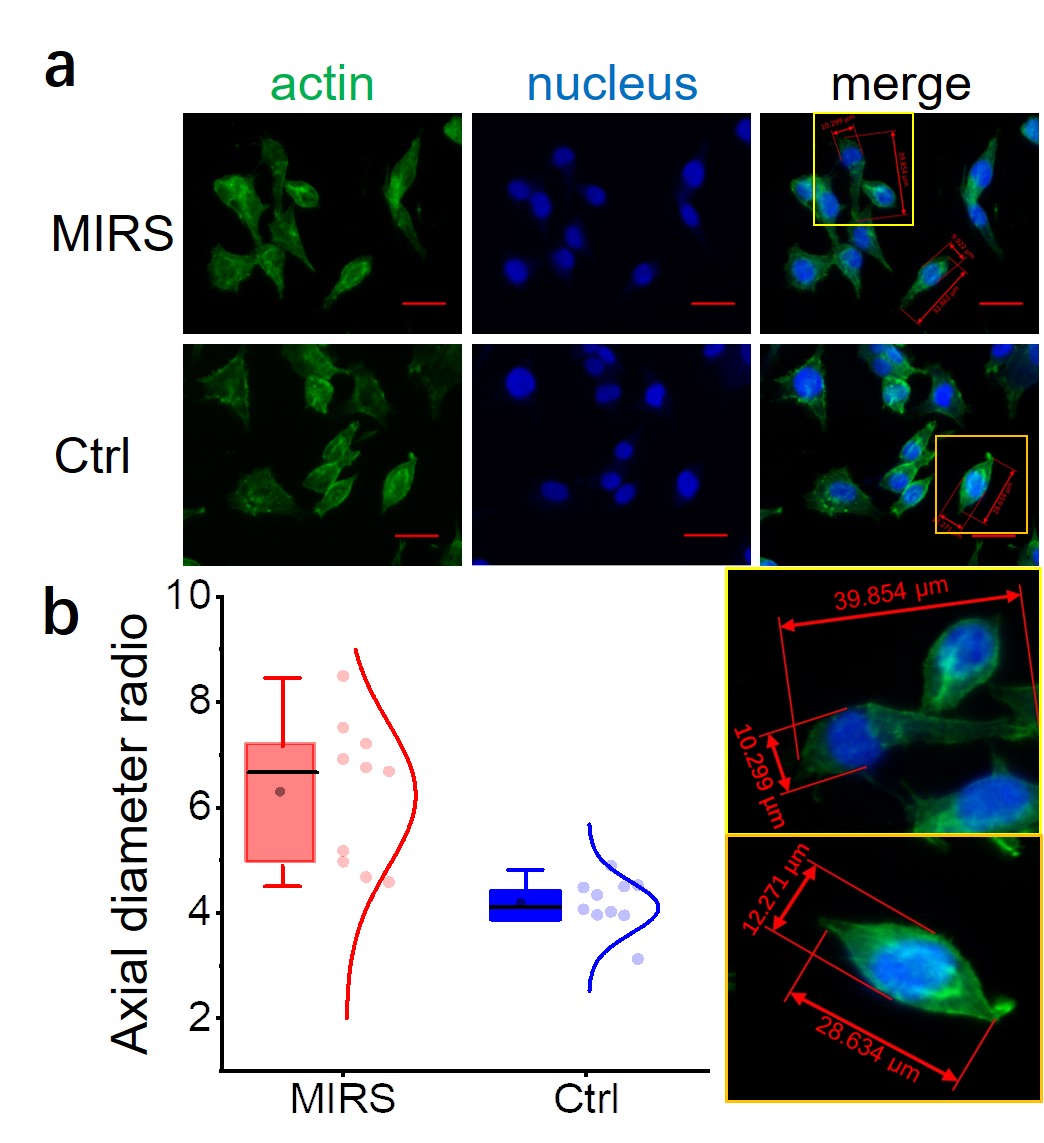


**Figure S5. MIRS enhanced fibroblast migration.** a) Morphological comparison between fibroblasts at 24 h with or without 1-min MIRS. Scale bar represents 20 μm. b) Comparison of cellular axial diameter ratio between fibroblasts with and without MIRS. The zoomed in pictures show the method for measuring the axial-diameter ratio.

**Figure S6. FTIR spectrum of the FP cavity.** The distance between optical frequency comb (peaks) is called the free spectral range (FSR), thereby the cavity distance can be determined.


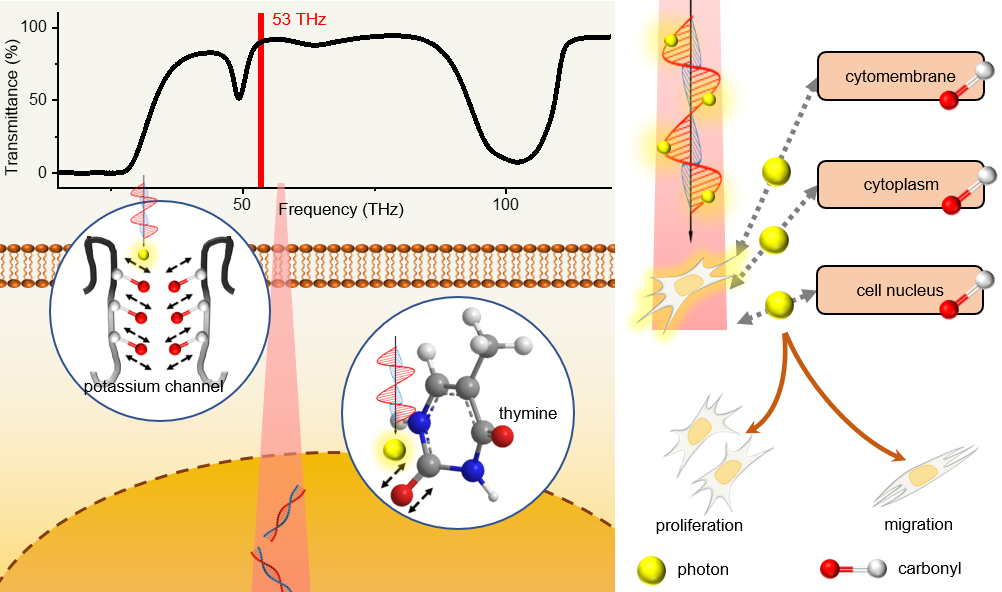


**Figure S7. Schematic of the nonthermal bioeffect of MIRS on fibroblasts.** Based on previous researches on the biological effects of carbonyl resonance between MIR and DNA bases and potassium channels, 5.6-μm MIR light was selected to check out the nonthermal bioeffects of resonant absorption on the electronic excited-state reactions within fibroblast cells. After 1-min MIRS, both the proliferation rate and migration ability of fibroblast cells were enhanced significantly.


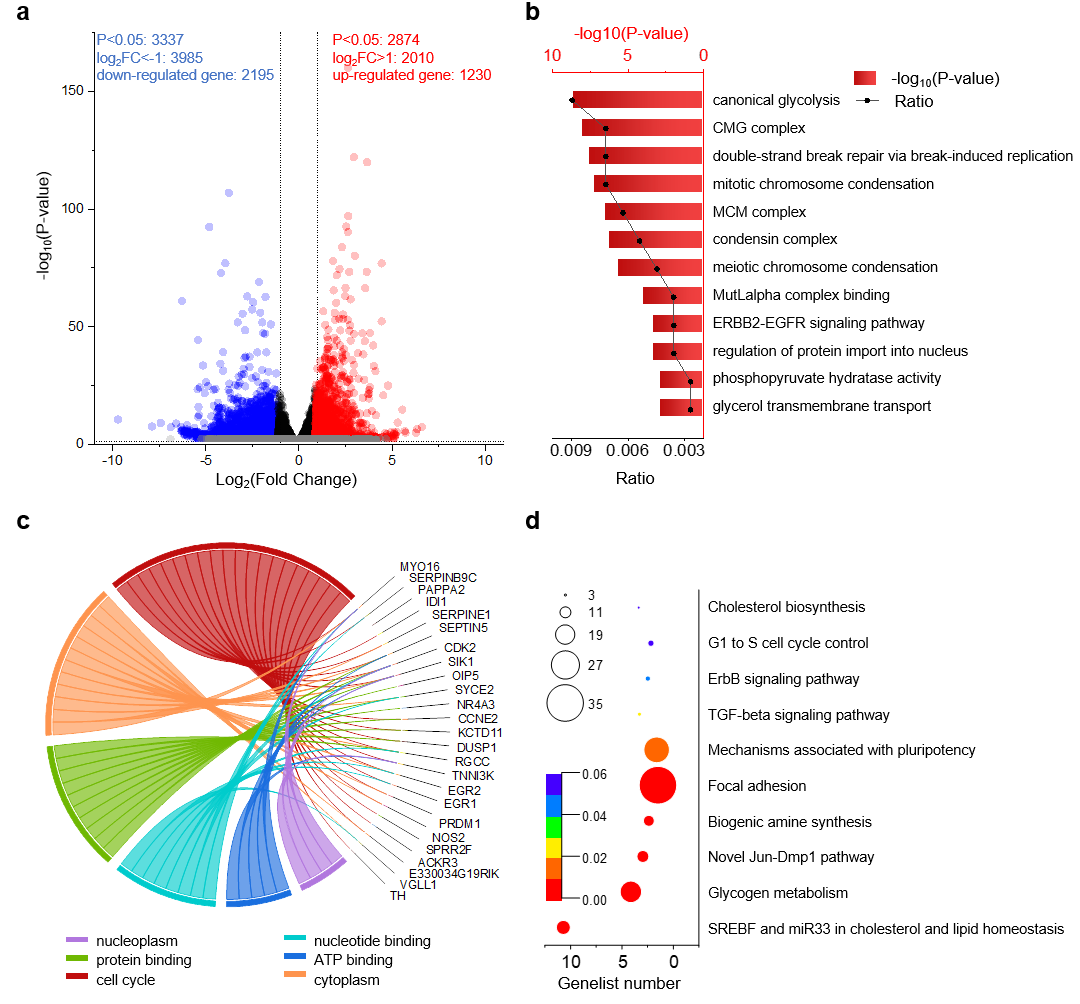


**Figure S8. Transcriptomic sequencing of fibroblasts at 24 h after VSC treatment­­­­­.** (a) Identification of significantly differentially expressed genes. (b) Gene ontology enrichment statistics, the histogram and plot line show the significance and the proportion of differentially expressed genes corresponding to each cell component term, respectively. (c) Chorography displays the interconnectivity between differentially expressed genes and GO enrichment terms. (d) Pathways that could be influenced by VSC. The color and size of bubbles represent the significance and the number of differentially expressed genes associated with the corresponding p­­athways, respectively.


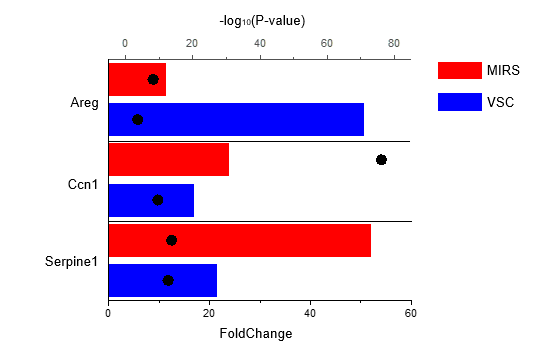


**Figure S9. Transcription of three important genes.** Transcriptional differences of Areg, Ccn1 and Serpine1 genes compared with control group at 24 h after MIRS and VSC treatments. The bar chart shows the difference multiple compared to the control group, and the dots represent the significance of the difference, expressed as -log_10_ (P-value).


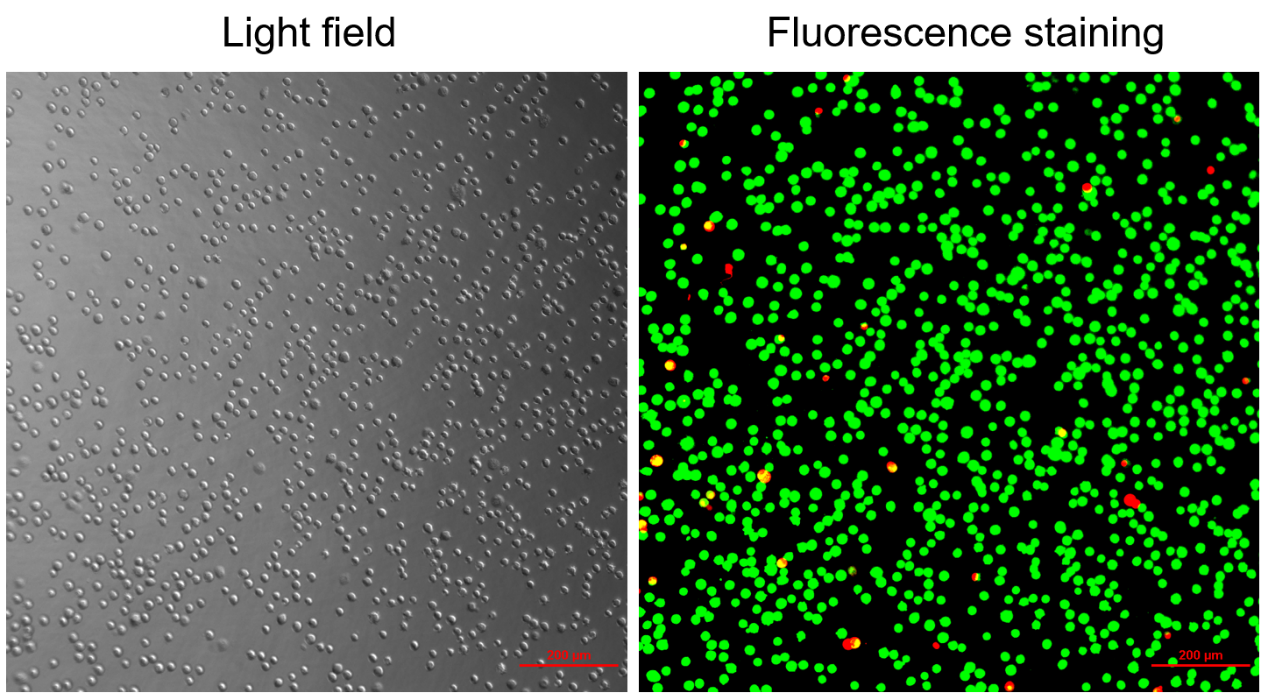


**Figure S10. Cell damage staining after FP cavity treatment.** Calcein-AM staining all living cells fluoresces green, while PI dye entering cells with membrane damage fluoresces red simultaneously. Scale bar represents 200 μm.


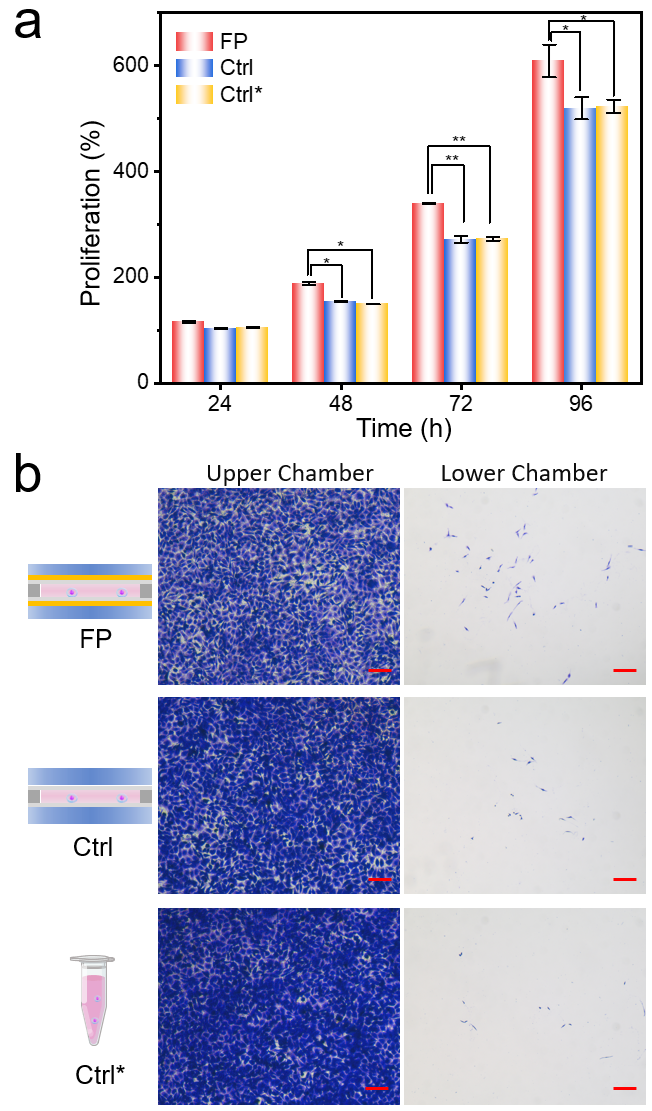


**Figure S11. Results of different control groups were compared in FP treatment experiment.** a) Comparison of cell proliferation rate at different culture time after FP cavity treatment. b) Results of cell transwell assay 48 h after FP cavity treatment. After FP chamber treatment, cells were inoculated into transwell chamber, and crystal violet staining images of upper and lower chambers were obtained 48 h later. Scale bar represents 100 μm.

**Supplementary Tables**

**Table S1. Primers used in qPCR**

| Gene Name | Size | Sequences |
| --- | --- | --- |
| mouse Areg | 110 | FP: GAAGACTCACAGCGAGGATGACAAG  RP: TGATAACGATGCCGATGCCAATAGC |
| mouse Serpine1 | 102 | FP: GTGCTGGTGAATGCCCTCTACTTC  RP: GACGGTGCTGCCATCAGACTTG |
| mouse Ccn1 | 90 | FP: ATGCGGTTCCGATGCGAAGATG  RP: GGGATGCGGGCAGTTGTAGTTAC |
